# Reciprocal regulation of IL-23 and IL-12 following co-activation of Dectin-1 and TLR signaling pathways

**DOI:** 10.1002/eji.200838543

**Published:** 2009-05

**Authors:** Kevin M Dennehy, Janet A Willment, David L Williams, Gordon D Brown

**Affiliations:** 1 Institute of Infectious Disease and Molecular Medicine, Clinical Laboratory Sciences Division of Immunology, University of Cape TownCape Town, South Africa; 2 Department of Surgery, James H. Quillen College of MedicineJohnson City, TN, USA

**Keywords:** Cell surface molecules, DC, Host/pathogen interactions, Innate immunity, Macrophages

## Abstract

Recognition of microbial products by germ-line-encoded PRR initiates immune responses, but how PRR mediate specific host responses to infectious agents is poorly understood. We and others have proposed that specificity is achieved by collaborative responses mediated between different PRR. One such example comprises the fungal β-glucan receptor Dectin-1, which collaborates with TLR to induce TNF production. We show here that collaborative responses mediated by Dectin-1 and TLR2 are more extensive than first appreciated, and result in enhanced IL-23, IL-6 and IL-10 production in DC, while down-regulating IL-12 relative to the levels produced by TLR ligation alone. Such down-regulation occurred with multiple MyD88-coupled TLR, was dependent on signaling through Dectin-1 and also occurred in macrophages. These findings explain how fungi can induce IL-23 and IL-6, while suppressing IL-12, a combination which has previously been shown to contribute to the development of Th17 responses found during fungal infections. Furthermore, these data reveal how the collaboration of different PRR can tailor specific responses to infectious agents.

## Introduction

Recognition of microbial products by germ-line-encoded PRR initiates host immune responses. The most intensively studied group of PRR, the TLR, recognize a widely expressed range of microbial structures 1. Given that most TLR signal through a common adaptor MyD88, it is unclear how recognition of these structures can translate into specific responses required for effective host defense. We and others have proposed that the collaboration of different PRR induces specific host immune responses 2,3. For example, co-ligation of TLR3, which couples to the adaptor TRIF (TIR-domain-containing adapter-inducing interferon-β) and not MyD88, with TLR7 synergistically induces IL-12 production and promotes development of Th1 responses 4. The β-glucan receptor, Dectin-1 and TLR2 are another PRR pair, which act synergistically, inducing the production of TNF 5.

Dectin-1 plays an essential role in the innate response to fungal pathogens 6. Recognition of fungal β-1,3-glucan by Dectin-1 can induce phagocytosis, the respiratory burst and the production of numerous cytokines and chemokines 7. Signaling through Dectin-1, which is largely mediated through Syk kinase, is thought to be sufficient for production of cytokines, such as IL-10, IL-6 and IL-23 7,8, whereas others, such as TNF, additionally require the recognition of another undefined fungal component by TLR2, and signaling through the MyD88 pathway 5. During fungal infection in mice, Dectin-1-mediated production of IL-23 and IL-6 promotes the development of Th17 responses 8. In line with this, human memory responses to *Candida albicans* were found to be predominantly Th17, and to a far lesser extent Th1 9. However, the mechanisms promoting such differential responses are not understood. We show here that although stimulation of Dectin-1 is sufficient for the production of IL-10, IL-6 and IL-23, co-ligation with TLR synergistically enhances these responses, while simultaneously down-regulating IL-12. These data provide a further example of how pairs or sets of PRR can tailor specific responses to infectious agents.

## Results

### Collaborative induction of IL-10 by the Dectin-1/Syk and TLR2/MyD88 pathways

**Figure 1:**
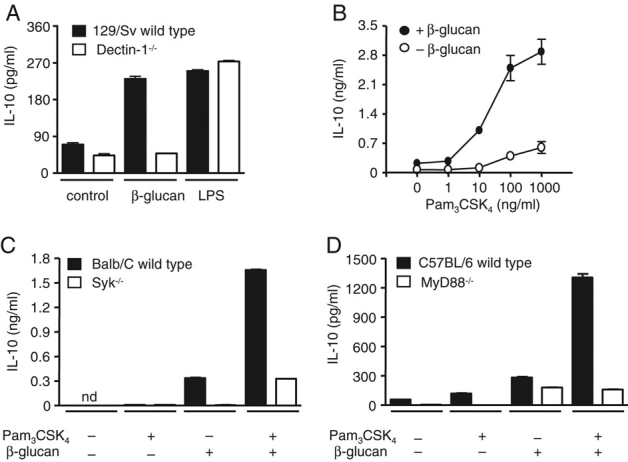
Collaborative induction of IL-10 by Dectin-1 and TLR2. (A) IL-10 production induced by 10 μg/mL β-glucan or 1 μg/mL LPS in 129/Sv WT and 129/Sv Dectin-1^−/−^ BMDC. (B) Production of IL-10 by C57BL/6 BMDC stimulated with the indicated concentrations of Pam_3_CSK_4_ with or without 10 μg/mL β-glucan. Induction of IL-10 in Balb/C WT and Syk^−/−^ (C) or C57BL/6 WT and MyD88^−/−^ (D) BMDC following stimulation with 10 μg/mL β-glucan and 10 ng/mL Pam_3_CSK_4_, as indicated. Data shown are mean±SD and are representative of two independent experiments.

Signaling from Dectin-1 is sufficient for the induction of cytokines, such as IL-10 10, but we speculated that these responses may also be influenced by collaborative signaling with TLR2, such as we have previously documented for the induction of TNF 5. For these experiments, we made use of highly purified ligands specific for each receptor 5,11 and determined the effect of stimulating Dectin-1 and/or TLR2 on IL-10 production in BM-derived DC (BMDC). As we reported previously 10, stimulation of these cells with purified β-glucan induced the production of IL-10 and this response was Dectin-1-dependent, as cells lacking this receptor did not respond to these carbohydrates (Fig. 1A). Both WT and Dectin-1^−/−^ BMDC produced comparable levels of IL-10 in response to LPS, demonstrating that β-glucan is a specific ligand in this system. Stimulation with TLR2-specific ligand, Pam_3_CSK_4_, also induced the production of IL-10 12, particularly at higher concentrations (Fig. 1B). However, combining this stimulus with β-glucan greatly enhanced IL-10 production, indicating that Dectin-1 and TLR2 were acting in a synergistic fashion for the induction of this cytokine (Fig. 1B).

Although β-glucan-induced IL-10 production is Syk-dependent 10, Dectin-1 can also signal *via* Syk-independent pathways 7, so we examined the downstream signaling components involved in the collaborative response, using BMDC from WT, Syk^−/−^ chimeric and MyD88^−/−^ mice. β-Glucan stimulated IL-10 production in a Syk-dependent fashion, as expected, but Syk-deficiency also ablated the synergistic response obtained with the co-addition of low concentrations of Pam_3_CSK_4_ (Fig. 1C). Similarly, synergistic IL-10 production was absent in MyD88^−/−^ DC, in which the response was comparable to that obtained following β-glucan stimulation alone (Fig. 1D). Thus, these results indicate that although Dectin-1 signaling is sufficient for the production of IL-10, co-stimulation of both the Dectin-1/Syk and TLR/MyD88 signaling pathways synergistically enhances this response.

### Co-ligation of Dectin-1 and TLR2 enhances IL-6 and IL-23 but down-regulates IL-12

**Figure 2:**
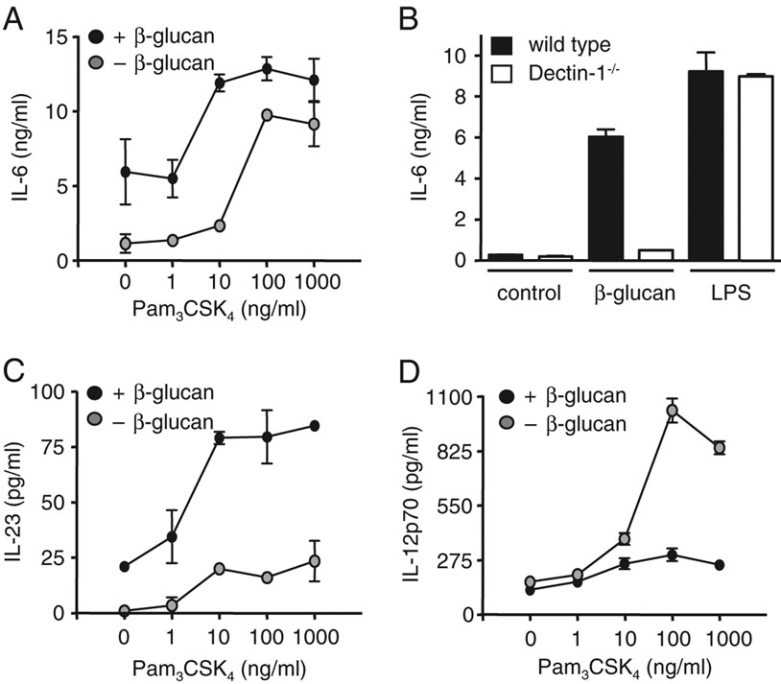
Co-ligation of Dectin-1 and TLR2 enhances production of IL-6 and IL-23 but down-regulates IL-12. (A) Production of IL-6 by C57BL/6 BMDC stimulated with the indicated concentrations of Pam_3_CSK_4_ with or without 10 μg/mL β-glucan. (B) IL-6 production induced by 10 μg/mL β-glucan or 1 μg/mL LPS in 129/Sv WT and Dectin-1^−/−^ BMDC. (C) Collaborative induction of IL-23 by C57BL/6 BMDC stimulated with the indicated concentrations of Pam_3_CSK_4_ with or without 10 μg/mL β-glucan. (D) Co-stimulation of C57BL/6 BMDC with the indicated concentrations of Pam_3_CSK_4_ and 10 μg/mL β-glucan down-regulates production of IL-12p70 relative to levels after Pam_3_CSK_4_ stimulation alone. Data shown are mean±SD and are representative of at least two independent experiments.

We then determined whether the production of other cytokines previously shown to be induced by Dectin-1 could also be influenced by co-stimulation with TLR2. We first examined IL-6 8, and found that the co-addition of β-glucan and Pam_3_CSK_4_ enhanced the production of this cytokine in a largely additive manner (Fig. 2A). As before, this response was dependent on Dectin-1 (Fig. 2B). β-Glucan stimulated the production of IL-23, as we have described previously 8, but this response could also be synergistically enhanced following co-ligation of TLR2 (Fig. 2C).

**Figure 3:**
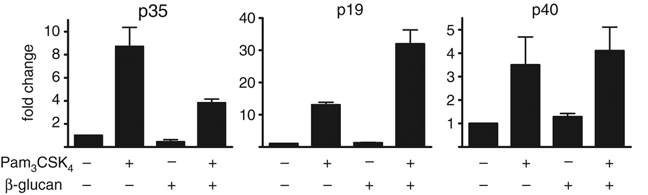
Co-ligation of Dectin-1 and TLR2 enhances IL-23p19 but down-regulates IL-12p35 at the transcriptional level. Real-time PCR data showing the relative levels of transcripts of IL-23p19, IL-12p35 and IL-12p40 following stimulation of Balb/C BMDC with 100ng/mL Pam_3_CSK_4_ and/or 10 μg/mL β-glucan, as indicated. Shown are mean±SEM of data pooled from two independent experiments.

**Figure 4:**
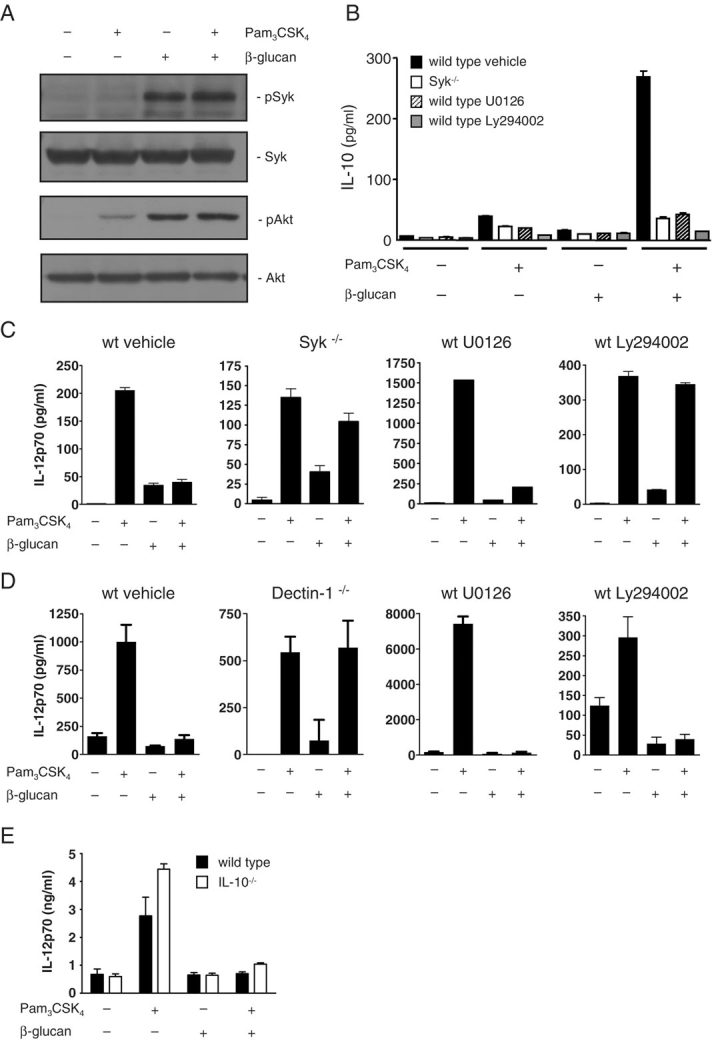
Down-regulation of IL-12 is dependent on signaling through Dectin-1. (A) Phosphorylation of Syk and Akt after stimulation of Balb/C BMDC with 10 μg/mL soluble β-glucan and 1 μg/mL Pam_3_CSK_4_, as indicated. Production of IL-10 (B) and IL-12p70 (C) from Balb/C Syk^−/−^ or WT thioglycollate-elicited macrophages pretreated with vehicle, 20 μM U0126 or 20 μM Ly294002, and stimulated with 10 μg/mL β-glucan and 10 ng/mL Pam_3_CSK_4_, as indicated. (D) Production of IL-12p70 from Balb/C WT or 129Sv Dectin-1^−/−^ BMDC pretreated with vehicle, 20 μM U0126 or 20 μM Ly294002, and stimulated with 10 μg/mL β-glucan and 100 ng/mL Pam_3_CSK_4_, as indicated. (E) Production of IL-12p70 from Balb/C WT or IL-10^−/−^ BMDC stimulated with 10 μg/mL β-glucan and 100 ng/mL Pam_3_CSK_4_, as indicated. Data shown are mean±SD and are representative of two independent experiments.

We also examined the induction of IL-12, in which Dectin-1 has been implicated 13, but in contrast to the other cytokines, we found that the production of IL-12 was paradoxically down-regulated relative to levels induced by TLR2 stimulation alone (Fig. 2D). As Dectin-1 is expressed on macrophages 14, we wondered if co-stimulation of TLR2 and Dectin-1 would also influence IL-12 production in these cells. Indeed, similar to that which we observed in DC, IL-12 was down-regulated in macrophages, relative to the level induced after TLR2 stimulation with Pam_3_CSK_4_ alone (data not shown, see Fig. 4). Consistent with all these observations, we found that co-stimulation of Dectin-1 and TLR2 on DC led to reduced levels of transcripts encoding p35, a subunit of IL-12p70, while simultaneously increasing the level of transcripts encoding p19, a subunit of IL-23 (Fig. 3A and B). By contrast, co-ligation of Dectin-1 did not markedly affect the levels of IL-12p40 transcripts (Fig. 3C). Thus co-stimulation of Dectin-1 and TLR2 enhances the production of IL-10, IL-6 and IL-23, but down-regulates the production of IL-12.

### Down-regulation of IL-12 requires signaling through Dectin-1 and is independent of IL-10

We next examined the mechanism of IL-12 down-regulation and investigated the roles of Syk kinase, ERK and PI3K. For this purpose, we first examined macrophages, which produce lower levels of IL-10 and IL-12 following stimulation through Dectin-1 alone, and hence give higher synergistic responses relative to the responses following ligation of Dectin-1 alone. Syk kinase is activated downstream of Dectin-1 10 (Fig. 4A), whereas activation of ERK occurs downstream of both Dectin-1 and TLR2 15,16. Activation of PI3K, indicated by Akt phosphorylation, can also be triggered by TLR2 17 and Dectin-1 18, but appears to occur primarily through Dectin-1 in this system (Fig. 4A). All of these kinases are known to regulate IL-10 16,19, and we could demonstrate that loss of Syk, or inhibition of ERK or PI3K prevented the synergistic production of IL-10 (Fig. 4B). By contrast, the inhibition of IL-12 production observed in WT macrophages after co-stimulation was largely lost in Syk-deficient macrophages (Fig. 4C).

Specific inhibition of PI3K with Ly294002 increased production of IL-12 following TLR2 stimulation alone (Fig. 4C and legend), consistent with previous reports that PI3K negatively regulates IL-12 production 20. However, in contrast to untreated macrophages, the levels of IL-12 were comparable after TLR2 stimulation and co-stimulation, suggesting that PI3K is required for IL-12 down-regulation. Inhibition of ERK with U0126 also increased the overall levels of IL-12 after TLR2 stimulation or co-ligation with Dectin-1, as expected 12,15, but the responses remained down-regulated after co-ligation relative to the response after TLR2 ligation alone (Fig. 4C and legend). Down-regulation of IL-12 in macrophages therefore appears to require both Syk and PI3K, but not ERK.

We next examined these responses in DC and, as we had observed in macrophages, inhibitors of these pathways blocked IL-10 production following co-ligation of Dectin-1 and TLR2 (data not shown). We could also demonstrate that IL-12 down-regulation in DC was mediated through Dectin-1, as this response was lost in Dectin-1-deficient cells (Fig. 4D). As before, inhibition of ERK with U0126 had no effect on IL-12 down-regulation; however, and in contrast to macrophages, the inhibition of PI3K with Ly294002 did not affect IL-12 down-regulation following receptor co-stimulation (Fig. 4D). These results therefore suggest that, although the mechanisms mediating this response are different, the down-regulation of IL-12 occurs in both of these cell types and is dependent on signaling induced through Dectin-1.

One possible mechanism of IL-12 down-regulation following receptor co-stimulation, is inhibition by the enhanced levels of IL-10 21. However, as the inhibition of ERK did not affect IL-12 down-regulation, despite abolishing IL-10 production, our results suggested that the effects on IL-12 were occurring independently of IL-10. To confirm this, we examined IL-12 production following co-stimulation of Dectin-1 and TLR2 in IL-10^−/−^ DC. As before, co-ligation of Dectin-1 and TLR2 in WT BMDC inhibited IL-12 production, relative to stimulation *via* TLR2 alone; however, IL-12 was also down-regulated in IL-10^−/−^ cells (Fig. 4E). Similar results were obtained with IL-10-deficient macrophages (data not shown). Thus IL-12 down-regulation following receptor co-stimulation occurs independently of IL-10.

### Down-regulation of IL-12 occurs upon co-ligation of Dectin-1 with multiple TLR

**Figure 5:**
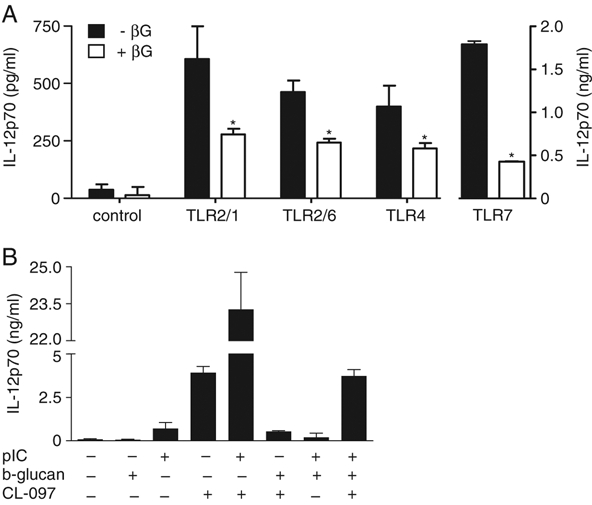
Down-regulation of IL-12 occurs following co-activation of Dectin-1 and multiple TLR. (A) Down-regulation of IL-12p70 following co-stimulation of thiogylcollate-elicited peritoneal macrophages with 10 μg/mL β-glucan (βG) and 100 ng/mL Pam_3_CSK_4_ (TLR2/1), 100 ng/mL FSL-1 (TLR2/6), 100 ng/mL LPS (TLR4) or 0.5 μg/mL CL-097 (TLR7), as indicated. ^*^p<0.05 (Student's *t*-test). (B) Production of IL-12p70 by Balb/C BMDC after stimulation with 0.5 μg/mL CL-097, 10 μg/mL poly(I:C) or 10 μg/mL β-glucan, as indicated. Data shown are mean±SD and are representative of two independent experiments.

We have previously shown that co-ligation of Dectin-1 with multiple MyD88-coupled TLR synergistically induces production of TNF 5 and we therefore determined if down-regulation of IL-12 also occurred under these conditions in macrophages. Indeed, we found that the addition of β-glucan down-regulated the production of IL-12 induced through multiple TLR, including TLR2/1, TLR2/6, TLR4 and TLR7 (Fig. 5A).

To further examine the effect of Dectin-1 on TLR responses, we then directly compared the induction of cytokines after co-ligation of TLR7 with either TLR3 or Dectin-1 in BMDC. We chose these PRR as collaboration of TLR7 with TLR3 has been previously shown to synergistically induce the production of IL-12 4. While co-ligation of TLR7 and TLR3 enhanced IL-12 responses, as expected, co-ligation of TLR7 and Dectin-1 down-regulated the production of IL-12 relative to that induced by TLR7 alone (Fig. 5B). Similarly, co-ligation of Dectin-1 down-regulated the high levels of IL-12 induced by the combination of TLR7 and TLR3 ligands. Thus Dectin-1 down-regulates the production of IL-12 induced through multiple TLR in both macrophages and DC.

## Discussion

The recognition of fungal pathogens, such as *C. albicans*, involves multiple receptors including several TLR and Dectin-1 22. We show here that co-ligation of Dectin-1 with multiple TLR down-regulates IL-12 production, while simultaneously enhancing production of other cytokines such as IL-10, IL-6 and IL-23.

Although induction of IL-10, IL-6 and IL-23 through Dectin-1 in DC has been demonstrated before 8,10, our data differ from previous findings in that we show that optimal IL-10, IL-23 and IL-6 production requires co-ligation of Dectin-1 and TLR, as opposed to ligation of Dectin-1 alone. Our data compliment recent studies that demonstrated that large particulate β-glucans and cytokines, such as GM-CSF, which is often present in BMDC cultures, enhances the responsiveness of Dectin-1 for production of particular cytokines 23,24. Indeed, the relatively high levels of IL-10, IL-23 and IL-6 observed previously 8,10 might be explained by the use of curdlan, a large particulate β-glucan, and the presence of GM-CSF in the culture medium. Our assays, in the absence of GM-CSF, show that co-ligation of TLR with Dectin-1 is required for optimal cytokine production. Thus, whereas previous studies have argued how single receptors influence responses, we demonstrate that pairs or sets of receptors collaborate to induce optimal cytokine responses.

As a second major finding of this paper, we show that PRR combinations, while inducing IL-10, IL-23 and IL-6, suppress the production IL-12. These results are similar to recent findings using human DC demonstrating that co-ligation of Dectin-1 with TLR2 and TLR7/8 similarly down-regulates IL-12 while up-regulating IL-23 25. However, in contrast to Gerosa *et al*. 25, we additionally show that Dectin-1 down-regulates IL-12 induced through a number of MyD88-coupled TLR (Fig. 5), and indeed even following stimulation with both TLR7 and TLR3 ligands which strongly up-regulate IL-12 (Fig. 5B). Because Gerosa *et al*. 25 do not observe down-regulation of IL-12 following co-ligation of Dectin-1 with any other TLR apart from TLR2, they suggest that such down-regulation may be due to signaling through TLR2. In contrast, we demonstrate that IL-12 down-regulation is mediated by Dectin-1, using Dectin-1-deficient cells (Fig. 4D). The considerable differences between our findings and those of Gerosa *et al*. 25 might be explained by the different species and stimulation conditions that were used. For example, murine BMDC were grown in GM-CSF which was removed prior to stimulation, whereas human monocyte-derived DC were grown in GM-CSF and IL-4 which were not removed prior to stimulation. Additionally, the ligands for Dectin-1 were different. We used a highly purified yeast-derived insoluble phagocytosible ligand, whereas Gerosa *et al*. 25 used a commercially available β-glucan, which has not been analyzed for purity. However, regardless of the differences between these two studies, the underlying concept remains that co-ligation of particular PRR can induce reciprocal regulation of IL-12 and IL-23. Indeed, fungal hyphae and the fungal cell wall preparation zymosan, which contain β-glucan and a number of TLR ligands, have been shown to induce high levels of IL-23 while concomitantly inducing low levels of IL-12 9,10,25. We argue that the reciprocal regulation of these cytokines that we observe here using pure ligands may explain how the production of these cytokines is regulated by fungi.

We have not defined a signaling mechanism by which IL-12 is down-regulated following co-ligation of Dectin-1 and TLR. However, we show in macrophages that such down-regulation requires both Syk and PI3K, which are activated downstream of Dectin-1. Recently it was shown that down-regulation of IL-12 in macrophages following co-ligation of FcγR and TLR4 also requires PI3K 19. Given that PI3K is predominantly activated downstream of FcγR in that system and Dectin-1 in our assays (Fig. 4A), the mechanism of IL-12 inhibition in macrophages may be similar. Moreover, down-regulation of IL-12 following co-ligation of FcγR and TLR4 occurs independently of IL-10 26, which we also observed following co-ligation of Dectin-1 and the TLR (Fig. 4E and data not shown). Thus the inhibition of IL-12 in macrophages occurs independently of IL-10 and mechanistically appears similar to that following TLR4 and FcγR ligation 19,26.

In contrast to macrophages, down-regulation of IL-12 following co-ligation of Dectin-1 and TLR in DC was not dependent on PI3K, ruling out a common mechanism in the two cell types. While it is clear that IL-12 down-regulation is dependent on the Dectin-1 pathway (Fig. 4D), the underlying mechanism in these cells remains an open question.

Lastly, our results demonstrating reciprocal regulation of cytokines following co-ligation of Dectin-1 and TLR are remarkably similar to recent reports demonstrating that co-ligation of NOD2 and various TLR down-regulates IL-12 production while concomitantly enhancing IL-23 production and promoting Th17 responses 27,28. Taken together with the present study, these results suggest that such reciprocal regulation of cytokines by sets of PRR may be a common mechanism to induce specific immune responses. Given that both Dectin-1 and NOD2 signal through the CARD9 (caspase recruitment domain 9) adaptor 29,30, it remains to be seen whether other CARD9-coupled receptors 31 similarly collaborate with TLR to promote Th17 responses.

## Materials and methods

### Reagents and mice

All TLR ligands were from InvivoGen (San Diego, CA), and the inhibitors U0126 and Ly294002 were from Calbiochem (Darmstadt, Germany). Production of highly purified particulate β-glucan and soluble β-glucan (glucan phosphate) have been described 11. Balb/C, Balb/C IL-10^−/−^, C57BL/6 and C57BL/6 MyD88^−/−^, 129/Sv and 129/Sv Dectin-1^−/−^ 6 mice were obtained from the animal unit of the University of Cape Town. Balb/C Syk^−/−^ chimeric mice were generated by the transfer of Syk^−/−^ fetal liver cells into irradiated Balb/C recipients, as described 5. All procedures were approved by the University of Cape Town animal ethics committee.

### Cell stimulation

Murine thioglycollate-elicited macrophages 32 were plated at 0.5–1×10^6^ cells/mL in RPMI 1640 medium containing 10% fetal calf serum (Gibco) and incubated overnight. The medium was replaced, and cells were stimulated with 10 μg/mL particulate β-glucan and 10 ng/mL Pam_3_CSK_4_, unless otherwise indicated, for 20 h. Immature murine BMDC were grown to >80% CD11c^+^MHCII^int^ as described 33, washed and incubated overnight in RPMI 1640 medium containing 10% fetal calf serum, plated at 4×10^5^ cells/mL and stimulated for 20 h. Cytokine secretion was assayed by ELISA using kits from Becton Dickinson (Mountain View, CA).

### Real-time PCR

BMDC were plated at 10^6^ cells/mL in RPMI 1640 medium containing 10% fetal calf serum (Gibco) and incubated overnight. Medium was replaced, and cells were stimulated with 10 μg/mL particulate β-glucan and 100 ng/mL Pam_3_CSK_4_ for 3 h. Cells were then lysed directly in Trizol LS (Invitrogen) and the RNA was further purified using RNeasy (Qiagen) before first strand cDNA synthesis using oligo dT primers and the ImProm-II Reverse Transcription System (Promega). Real-time PCR using LightCycler FastStart DNA Master PLUS SYBR Green kit (Roche) was performed using primers for IL-12a p35 (QuantiTect primer QT01048334, Qiagen), IL-12b p40 (QuantiTect primer QT00153643, Qiagen), IL-23 p19 34 and ribosomal protein S12 (forward 5′-GGAAGGCATAGCTGCTGGAGG-3′ and reverse 5′-CGATGACATCCTTGGCCTGA-3′; a kind gift of Dr. Anita Schwegmann, University of Cape Town). Relative mRNA expression values were obtained by dividing the calculated transcript levels by the rS12 levels for each sample, and results are presented as fold change in transcript levels *versus* untreated controls.

### Syk and akt phosphorylation

BMDC (10^7^ cells in 100 μL HBSS) were stimulated with 10 μg/mL soluble β-glucan and 1 μg/mL Pam_3_CSK_4_ for 3 min at 37°C before addition of lysis buffer (1% NP40, 25 mM Tris pH 8, 10 mM EDTA, 140 mM NaCl, 5 mM NaF, 1 mM Na_3_VO_4_, 5 mM iodoacetamide) containing protease inhibitors (Roche). Lysate supernatants were resolved by SDS-PAGE, Western-blotted and probed with antibodies to phospho-Syk and Syk or phospho-Akt (Ser473) and Akt (Cell Signaling, MA).

## Abbreviation

BMDC: BM-derived DC
